# Hemodynamic response to non-pneumatic anti-shock compression garments in patients with renal dysfunction

**DOI:** 10.1186/s12882-019-1680-8

**Published:** 2020-01-14

**Authors:** R. Marinovich, Z. Li, T. Tamasi, K. Quinn, S. Wong, C. W. McIntyre

**Affiliations:** 1 Schulich School of Medicine, Medical Sciences Building Room M101, Western University, London, ON N6A 5C1 Canada; 2 Kidney Clinical Research Unit, London Health Sciences Centre, 800 Commissioners Rd E, London, ON N6A 5W9 Canada

**Keywords:** Compression, Chronic kidney disease, Dialysis, Hemodynamics, Cardiovascular

## Abstract

**Background:**

Patients with chronic kidney disease are at higher risk of developing cardiovascular disease. Chronic exposure to intermittent hemodialysis may be a source of added stress to the cardiovascular system; intradialytic hypotension is a common complication of hemodialysis, and repeated events may lead to hemodynamic stress and ischemic injuries. Administration of non-pneumatic compression stockings to the lower limbs has demonstrated hemodynamic stabilizing effects in other settings and may provide similar benefits in the kidney disease population. Therefore, we conducted this pilot study assessing the feasibility and tolerability of the application of non-pneumatic compression stockings to patients with kidney disease. We also assessed the changes in hemodynamic measurements following the application of the compression stockings to explore the biological feasibility of this being an effective intervention for intradialytic hypotension.

**Methods:**

Fifteen individuals were enrolled in the study (5 healthy, 5 chronic kidney disease patients, and 5 dialysis patients). Outcomes including hemodynamic parameters such as cardiac output, peripheral vascular resistance, and blood pressure were measured using continuous pulse wave analysis. Changes in global longitudinal strain were measured via echocardiography. These outcome measurements were made before and after the application of compression stockings.

**Results:**

All study participants tolerated the compression garments well and without complication. Hemodynamic response to lower body compression caused varying effects on cardiac output, mean arterial pressure and global longitudinal strain. Some individuals saw large improvements in hemodynamic parameters while in others the opposite effect was observed. No consistent response was elicited.

**Conclusions:**

Application of compression stockings to patients with renal dysfunction is well-tolerated. However, significant variations in hemodynamic outcomes exist, and may be a barrier for larger scale trials without prior identification of specific patient characteristics indicating likely benefit from the application of external compression.

**Trial registration:**

ClinicalTrials.gov, Identifier: NCT02915627, Registration Date: Sept 27, 2016.

## Background

It is well recognized that patients with end stage renal disease (ESRD) requiring intermittent hemodialysis (HD) suffer excess cardiac morbidity and mortality [1]. There are disproportinaltely higher rates of heart failure and cardiac arrhythmia [2] in this population and cardiovascular disease remains the leading cause of mortaliy. In addition to the traditional cardiovascular risk factors, intra-dialytic hypotension (IDH) may be a key contributor to myocardial injury, and repeated injury during routine dialysis further precipitate progression of cardiovascular deterioration. For instance, previous investigations have found evidence of reduction in myocardial blood flow during dialysis, resulting in ischemia and myocardial stunning. Over time, such repeated injury can lead to ventricular remodeling that may further contribute to hemodynamic instability and dysregulation [3, 4].

Intra-dialytic hypotension is one of the most common complication of HD treatment, occurring in approximately 20–30% of HD sessions [5]. Clinically, IDH often leads to premature cessation of dialysis sessions, or decreased fluid removal, both of which not only decrease dialysis efficiency and effect, but also leads to decreased quality of life. Furthermore, IDH has been associated with other medical complications such as vascular access thrombosis, and mesenteric ischemia [6]. Current treatment strategies for IDH are aimed to address the rate of fluid shift through altering the ultrafiltration rate and dialysate electrolyte concentrations. Vasoconstrictive medications such as midodrine are commonly used with some success, however, effect tends to diminish over time and their safety has been called into question [7]. Novel strategies are needed to further address this issue.

One such approach to the management of IDH is mechanical augmentation via the application of lower limb compression stockings during dialysis [8–10]. These devices increase venous return and contributes to additional intravascular volume, preload, and in turn, cardiac output. In particular, the non-pneumatic anti-shock compression garments have been used since the early 1900s to manage shock [11]. Benefit has been demonstrated in patients with hypovolemic shock, such as traumatic injury, abdominal aortic aneurysm rupture, and postpartum hemorrhage; and in distributive shock, such as in the setting of anaphylaxis [11]. In addition to improving cardiac pre-load, when applied to the abdomen, the anti-shock garments may exert hormonal effects via sympathetic stimulation, and further improve blood pressure [12]. In the dialysis population, the benefit of compression stockings is conflicting. Two studies have examined the benefit of pneumatic compression stockings: a randomized crossover study by Tai et al. did not find significant benefit [13], while Alvares et al. [14], also using a randomized crossover design, did find a reduction in IDH with the use of pneumatic compression in the first hour of dialysis. However, the use of non-pneumatic devices remains un-explored.

In contrast to pneumatic compression stockings which provide thigh high intermittent compression, non-pneumatic anti-shock compression garments apply continuous pressure to not only the lower limbs but also upper thigh and abdomen. The ZOEX Non-Inflatable Anti-Shock Garment (NASG) is a specific type of anti-shock garment originally developed from NASA anti-G-force technology which received FDA device approval for management of haemorrhagic shock in 1991. They are a lightweight neoprene garment that is made up of six segments that close tightly with Velcro. This garment is worn on the lower extremities (legs) and applies 20–40 mmHg pressure to the lower body and abdomen.

The utility of NASG to raise systemic blood pressure is already well established through work done on obstetrical haemorrhages in remote locations. Two independent groups working in Pakistan report a marked elevation in systolic blood pressure within minutes of application of NASG to patient suffering from obstetrical haemorrhage [15, 16]. Later prospective work by Miller et al. demonstrated a decrease in both blood loss, morbidity and mortality in patients with postpartum bleeding using NASG [17–19]. Furthermore, this work demonstrated that the use of NASG over extended periods is safe. Over a 36-h period of continuous use, no adverse events were reported in the study group [17–19]. The immediacy in which the Zoex NASG modulates hemodynamic variables combined with the lack of adverse effects even over extended use makes this device promising for use in the dialysis setting. The proven benefit in other settings, the relatively low cost, and the ease of application, make these devices a worthwhile option for further exploration.

We therefore conducted this pilot study to examine the effects of non-pneumatic compression garments on individuals with varying degree of renal dysfunction. Through this small and focused trial. We aim 1) to assess the tolerability of these garments, 2) to examine the hemodynamic effects of these compression garments through continuous hemodynamic monitoring using the Finapres technology, and 3) to assess the feasibility for potential larger scale clinical trials.

## Methods

### Study design

In this prospective, interventional, pilot study, non-pneumatic anti-shock compression garments were applied to individuals with varying degrees of renal disease. Three groups of participants were analyzed: 1) Healthy participants with no known kidney disease which served as a control group, 2) participants with stage 4 or 5 chronic kidney disease (CKD) not receiving dialysis, and 3) individuals with end stage renal disease currently receiving intermittent hemodialysis.

### Participants

Five patients were recruited for each arm of the study. Healthy participants were recruited via poster board advertisement at London Health Science Centre (LHSC), Victoria Campus, London, Ontario, while CDK patients were recruited from the LHSC CKD clinic based at a community dialysis site. Dialysis patients were recruited from the Victoria Hospital dialysis unit population, also at LHSC.

### Inclusion and exclusion criteria

All participants were required to be over the age of 18 and were of both sexes. Healthy participants had no clinical diagnosis of CKD. Non-dialysis CKD patients were required to have diagnosed stage 4 or 5 CKD but were not receiving and had never had any form of dialysis. Dialysis patients were required to be undergoing active intermittent hemodialysis at the time of the study protocol. Participants were excluded if they had New York Heart Association Class IV heart failure, absence of lower limbs or lower limb injury, or individuals with established peripheral vascular disease or clear symptoms of claudication. Additionally, patients who declined participation, were pregnant, or did not meet inclusion criteria were excluded.

### Compression garments

ZOEX Non-Inflatable Anti-Shock Garment (NASG), supplied by ZOEX NIASC, Coloma, CA, USA were used for all participants of this study. They are a compression garment consisting of 6 segments that close tightly with Velcro that applies a continuous pressure of 20–40 mmHg to the lower limbs and abdomen.

### Finometer

The use of the Finometer (Finapres Medical Systems, Arnhem, Netherlands) in dialysis patients has been described in detail elsewhere [20] and was used to monitor a variety of hemodynamic parameters, including cardiac output and mean arterial pressure, during the study protocol.

### Study protocol

On the day of the study visit, participants were subjected to a basic history review and a baseline set of routine vitals were taken to calibrate the Finometer. Patients were weighed, and their current weight was compared against their ideal weight as an estimate of fluid balance (Additional file 1: Table S1). Participants were laid supine on an examination table with the Finometer attached and without compression garments on. Baseline data for each participant was collected for a total of 15 min. Five minutes into the session a baseline echocardiogram was performed. Following this first interval, participants donned the compression garments and were again laid supine on an examination table. Data was again collected for 15 continuous minutes with an echocardiogram being performed 5 min after application of the compression garments. For dialysis patients, the study visit occurred on a day they would normally receive dialysis, but before their dialysis session.

### Regarding statistical analysis

This pilot study was designed to characterize the hemodynamic of patients with varying degrees of renal dysfunction in the presence and absence of lower body compression. This study was not sufficiently powered to show an effect on cardiac output or mean arterial pressure with the application compression garments and as such a statistical analysis to that end was not performed. Descriptive and summary statistics were computed with Microsoft Excel 2018.

## Results

### Patients

Baseline characteristics for study participants are presented in Table 1. Significant heterogeneity was observed between individuals within groups, particularly with regards to their medications and comorbidities. Healthy participants were significantly younger than both CKD and dialysis participants and represent a cardiovascularly healthy optimal control.

**Table 1:** Baseline characteristics for study participants, each arm contained five patients

Group	Dialysis (*n* = 5)	CKD (*n* = 5)	Healthy (*n* = 5)
Average Age	66.3	74.4	46.8
Causes for CKD (n)			
Diabetes Mellitus	4	1	N/A
Baseline SBP (mmHg)	136.5	155.8	135.8
Baseline DBP (mmHg)	83.5	81.4	88.4
Baseline HR (bmp)	94.5	73	72.6
Medications (n)			
Beta-Blocker	1	2	0
Calcium Channel Blocker	1	3	0
Diuretic	0	2	0
ACE inhibitor/ARB	0	1	0
MRA	0	1	0
Cardiovascular Comorbidities (n)			
CAD	1	0	0
Hypertension	4	4	0
Atrial Fibrillation	4	0	0

Note: *CKD* Chronic kidney disease, *SBP* Systolic blood pressure, *DBP* Diastolic blood pressure, *HR* Heart rate, *ACE* Angiotensin converting enzyme, *ARB* Angiotensin receptor blocker, *MRA* Mineralocorticoid receptor antagonist, *CAD* Coronary artery disease

### Hemodynamic response

**Fig. 1:**
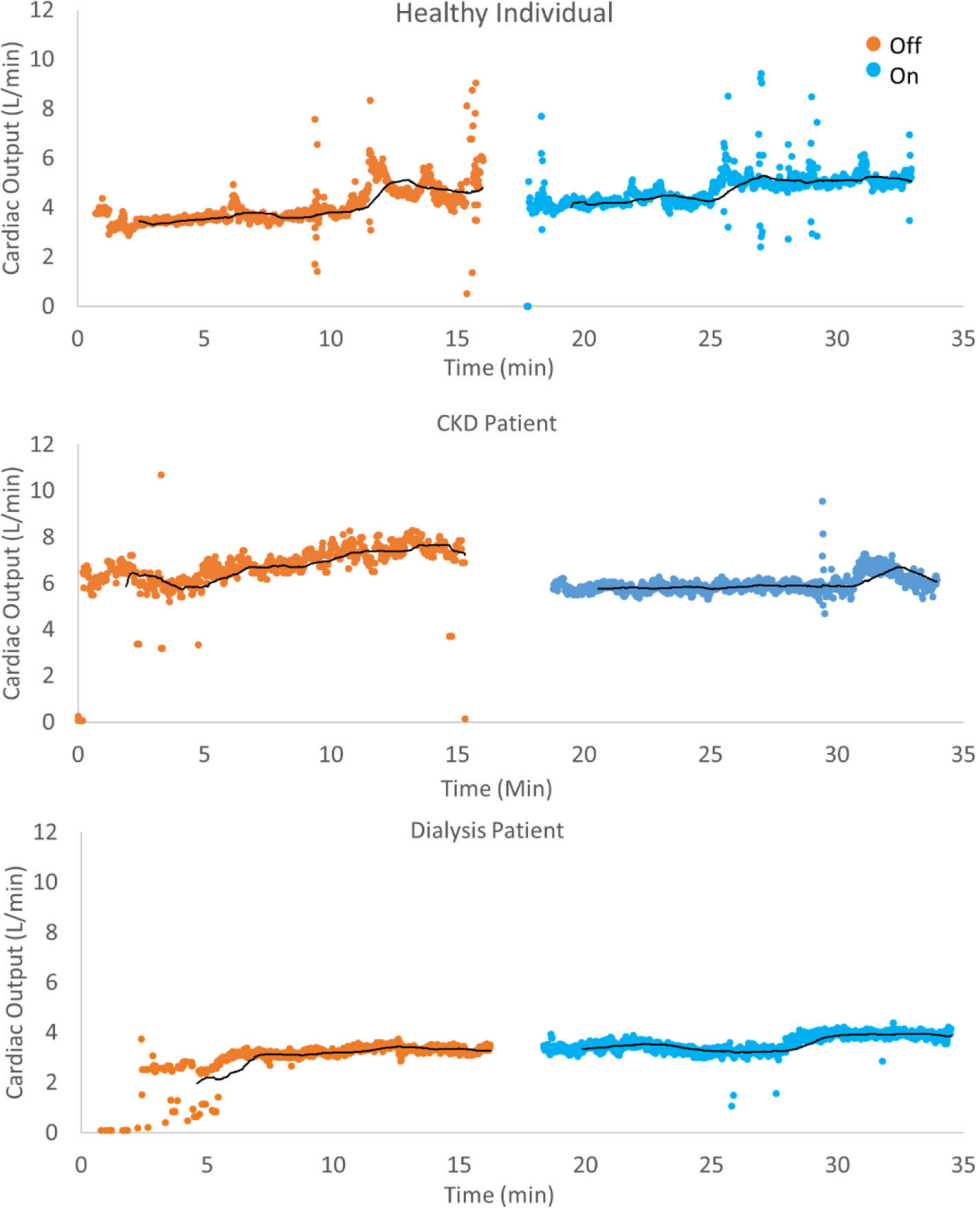
Representative data series of cardiac output from individual patients of each cohort. Baseline characteristics in approximately 15-min intervals displayed in orange. Application of non-pneumatic compression stocks occurred subsequently, and hemodynamic response is shown in blue

**Fig. 2:**
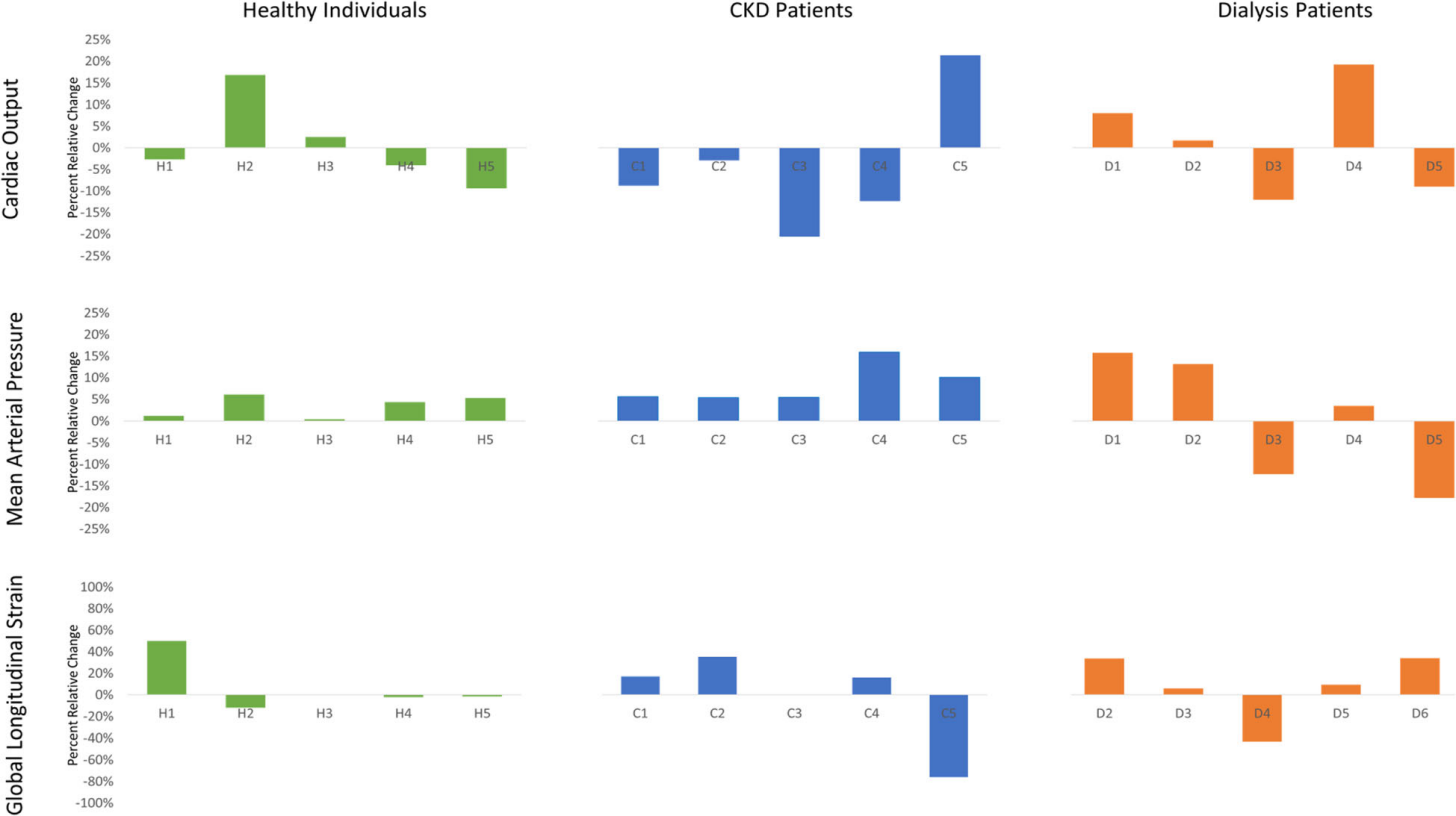
Hemodynamic response of patients with kidney disease to non-pneumatic compression stockings. The relative change in cardiac output, mean arterial pressure and global longitudinal strain after the application of non-pneumatic compression stockings in heathy individuals, CKD patients not on dialysis, and dialysis patients are displayed. Cardiac output and mean arterial pressure data were collected via Finometer while global longitudinal strain was obtained via echocardiography

Figure 1 illustrates the continuous cardiac output of a representative individual from each group over the course of the study procedure. Mild fluctuations in cardiac output are seen throughout in each patient, however a stable baseline is readily apparent. No obvious or reproducible changes in cardiac output were observed over the 15-min interval in which the compression garments were worn, indicating that any hemodynamic effect caused by application of the garments was immediate and sustained. Similar scatter plots were generated for each study participant for several hemodynamic parameters. Individual responses to the compression garments were quite variable (Fig. 2, Table 2) and no clear or significant trend was observed in any group with the exception of CKD participants who uniformly experienced an increase in mean arterial pressure when wearing the compression garments.

**Table 2:** Hemodynamic response to non-pneumatic compression stockings. Hemodynamic parameters from healthy individuals (H), CKD patients (C) and participants receiving hemodialysis (D) are displayed. Data is related as a percent change from baseline (without compression garments)

Group	Heart Rate	Stroke Volume	Cardiac Output	Peripheral Resistance	Systolic Pressure	Mean Arterial Pressure	Diastolic Pressure
H1	−0.9%	−1.7%	−2.7%	5.8%	1.0%	1.3%	2.7%
H2	−3.3%	20.2%	16.8%	−13.1%	9.6%	6.1%	2.7%
H3	1.1%	−1.5%	2.5%	4.7%	1.5%	0.4%	0.5%
H4	−8.4%	−3.2%	−4.1%	14.5%	3.2%	4.3%	6.1%
H5	4.2%	−13.0%	−9.4%	21.8%	2.8%	5.3%	8.1%
Mean	−1.5%	0.1%	0.6%	6.7%	3.6%	3.5%	4.0%
St. Dev.	4.8%	12.0%	10.0%	13.0%	3.0%	3.0%	3.0%
C1	8.2%	−10.6%	−8.8%	17.8%	6.2%	5.6%	8.6%
C2	−0.5%	−3.2%	−3.0%	11.6%	3.5%	5.4%	4.9%
C3	−1.0%	−19.8%	−20.6%	47.1%	−2.3%	5.5%	11.4%
C4	14.5%	−16.5%	−12.4%	43.8%	8.9%	15.9%	16.1%
C5	4.4%	16.0%	21.3%	−11.1%	15.1%	10.1%	5.5%
Mean	5.1%	−6.8%	−4.7%	21.8%	6.3%	8.5%	9.3%
St. Dev.	6.5%	14.2%	15.9%	24.1%	6.4%	4.6%	4.6%
D1	−3.0%	11.4%	8.0%	8.6%	21.0%	15.7%	14.1%
D2	−3.4%	3.0%	1.7%	10.4%	11.5%	13.1%	9.0%
D3	−3.0%	−7.8%	−12.0%	0.1%	−16.9%	−12.3%	−9.9%
D4	−3.8%	17.7%	19.2%	−19.2%	4.6%	3.4%	2.7%
D5	8.4%	−15.7%	−9.1%	−12.5%	−22.4%	− 17.8%	−15.9%
Mean	−0.9%	1.7%	1.5%	−2.5%	−0.4%	0.4%	0.0%
St. Dev.	5.3%	13.6%	12.7%	13.0%	18.6%	15.0%	12.6%

## Discussion

In this pilot study we assessed the tolerability and hemodynamic response of a small group of patients with varying degrees of kidney disease to the application of non-pneumatic anti-shock compression garments. In general, the compression garments were well tolerated by all study participants, however, individuals with pre-existing peripheral vascular disease were noted to most likely decline participation in the trial. We performed continuous hemodynamic monitoring via the Finapres system and obtained multiple cardiac parameters. Interestingly, despite complete and robust data collection from each patient, no consistent trend was observed within or between groups of varying renal function. However, there seem to be greater degree of variability in the CKD and HD groups, as compared to the healthy group.

The cause of the variability in response to compression garments is likely multifactorial. In this small scale study, there is significant heterogeneity in participant’s characteristics. Underlying medical comorbidity, cardiovascular health, and hemodynamic modifying mediations are among some of the factors that have likely contributed to the response of each individual in our study. For instance, in the HD group, the experiments were conducted prior to dialysis sessions for ease of participant scheduling. As a result, these participants may be relatively hypervolemic, and augmentation of preload in this state may have diminished benefit, or even detrimental effect. Therefore, the lack of consistent response may simply reflect the variable fluid status in these individuals. Patient fluid balance as estimated by change from the patient’s ideal weight (Additional file 1: Table S1) did not appear to be related to the hemodynamic response observed. Furthermore, the higher degree of variability between individuals was not unexpected as the increase in pre-load may be substantial, and different responses may reflect underlying cardiovascular fitness and right heart function.

In comparison between groups, our qualitative observation of higher degrees of hemodynamic variability in the two groups with renal dysfunction may be at least partly explained by impaired autonomic regulation, and inability to respond to the increased pre-load in these populations. Another source of variability is the concurrent use of beta blockers or renin-aldosterone system blockade agents which may further impair this regulation system [21], leading to additional sources of variability.

In light of our data, large scale studies such as the study by Tai et al. may not observe a significant benefit statistically as the underlying variability due to patient heterogeneity may dilute the true effect. As such, study of generalized application of these compression stockings may not be the best approach. Our study highlights the need for further characterization of factors that would predict beneficial response. For instance, compression garments may be more suitable to individuals with certain known echocardiographic features, or application of these garments may be reserved on an as-needed basis, rather than universal application. However, in clinical setting, this may be difficult, as clinical variables such as intravascular volume status is difficult to determine accurately, especially since this variability naturally exist between individuals and within each individual across time. Therefore, such tailored but restricted approach may not be pragmatic in a clinical setting.

Our study is limited in its small size, and descriptive in nature. However, our protocol reflected the pragmatic approach that would be applied in a large scaled study, and the smaller sample size allowed for mechanistic assessment of the intervention. Despite excellent tolerability, we found significant variation in hemodynamic response. This inconsistency calls into questions the likely utility of universal application of our compression garments to patients with renal dysfunction and informs that large scale study based on similar a protocol will likely result in statistically negative trial. Our results provide mechanistic insights into the use of these garments and poses questions for future research.

## Conclusions

Non-pneumatic anti-shock compression garments inexpensive and readily available devices that are tolerable and appear feasible to utilize in the CKD and dialysis setting from a practical perspective. We have also highlighted the significant heterogeneity of hemodynamic responses to lower body compression in patients with renal dysfunction, which may explain previous studies’ failure to show benefit with generalized application of these garments. Further study is warranted to refine patient selection and identify candidate who would be most likely to receive benefit from nonpneumatic compression garments and we remain optimistic for novel application methods of these devices.

## Supplementary information

**Additional file 1: Table S1.** Study participants fluid balance as estimated by change from ideal weight. CKD participants (C) and dialysis participants (D) were weighted on the day of study. This weight was compared to their ideal weight, obtained from their recent nephrology clinic notes. The difference between study weight and ideal weight is intended to provide an estimate to the fluid balance of the study participant on the day of study.

## Data Availability

The datasets used and/or analyzed during the current study are available from the corresponding author on reasonable request.

## Abbreviations

ACE: Angiotensin converting enzyme
ARB: Angiotensin receptor blocker
CAD: Coronary artery disease
CKD: Chronic kidney disease
DBP: Diastolic blood pressure
ESRD: End stage renal disease
HD: Hemodialysis
HR: Heart rate
IDH: Intra-dialytic hypotension
LHSC: London Health Science Centre
MRA: Mineralocorticoid receptor antagonist
NASG: Non-inflatable anti-shock garment
SBP: Systolic blood pressure

## References

[1] Foley RN, Parfrey PS, Sarnak MJ (1998). Clinical epidemiology of cardiovascular disease in chronic renal disease. Am J Kidney Dis.

[2] Kalantar-Zadeh K, Regidor DL, Kovesdy CP, Van Wyck D, Bunnapradist S, Horwich TB (2009). Fluid retention is associated with cardiovascular mortality in patients undergoing long-term hemodialysis. Circulation..

[3] Burton JO, Jefferies HJ, Selby NM, McIntyre CW (2009). Hemodialysis-induced cardiac injury: determinants and associated outcomes. Clin J Am Soc Nephrol.

[4] McIntyre CW, Burton JO, Selby NM, Leccisotti L, Korsheed S, Baker CSR (2008). Hemodialysis-induced cardiac dysfunction is associated with an acute reduction in global and segmental myocardial blood flow. Clin J Am Soc Nephrol.

[5] Bos WJ, Bruin S, van Olden RW, Keur I, Wesseling KH, Westerhof N (2000). Cardiac and hemodynamic effects of hemodialysis and ultrafiltration. Am J Kidney Dis.

[6] Flythe JE, Xue H, Lynch KE, Curhan GC, Brunelli SM (2015). Association of mortality risk with various definitions of intradialytic hypotension. J Am Soc Nephrol.

[7] Brunelli SM, Cohen DE, Marlowe G, Van Wyck D (2018). The impact of Midodrine on outcomes in patients with Intradialytic hypotension. Am J Nephrol.

[8] Ahsan M, Gupta M, Omar I (2004). Prevention of hemodialysisrelated muscle cramps by intradialytic use of sequential compression devices: a report of four cases. Hemodial Int.

[9] Beninson J, Levin NW, Santiago G (1974). Use of intermittent pneumatic compression in hemodialysis. Proc Clin Dial Transplant Forum.

[10] Onuigbo MA (2010). Bilateral lower extremity sequential compression devices (SCDs): a novel approach to the management of intradialytic hypotension in the outpatient setting–report of a case series. Ren Fail.

[11] Lateef F, Kelvin T (2008). Military anti-shock garment: historical relic or a device with unrealized potential?. J Emerg Trauma Shock.

[12] Garvin NM, Levine BD, Raven PB, Pawelczyk JA (2014). Pneumatic antishock garment inflation activates the human sympathetic nervous system by abdominal compression. Exp Physiol.

[13] Tai DJ, Ahmed SB, Palacios-Derflingher L, Hemmelgarn BR, JM MR (2013). Alberta kidney disease network. Pneumatic compression devices during hemodialysis: a randomized crossover trial. Nephrol Dial Transplant.

[14] Álvares VRC, Ramos CD, Pereira BJ, Pinto AL, Moysés RMA, Gualano B, Elias RM (2017). Pneumatic compression, but not exercise, can avoid Intradialytic hypotension: a randomized trial. Am J Nephrol.

[15] Brees C, Hensleigh PA, Miller S, Pelligra R (2004). A non-inflatable anti-shock garment for obstetric hemorrhage. Int J Gynaecol Obstet.

[16] Hensleigh PA (2002). Anti-shock garment provides resuscitation and haemostasis for obstetric haemorrhage. BJOG..

[17] Miller S, Ojengbede O, Turan JM, Morhason-Bello IO, Martin HB, Nsima D (2009). A comparative study of the non-pneumatic anti-shock garment for the treatment of obstetric hemorrhage in Nigeria. Int J Gynaecol Obstet.

[18] Miller S, Martin HB, Morris JL (2008). Anti-shock garment in postpartum haemorrhage. Best Pract Res Clin Obstet Gynaecol.

[19] Miller S, Fathalla MMF, Ojengbede OA, Camlin C, Mourad-Youssif M, Morhason-Bello IO (2010). Obstetric hemorrhage and shock management: using the low technology non-pneumatic anti-shock garment in Nigerian and Egyptian tertiary care facilities. BMC Pregnancy Childbirth.

[20] SH SNML, Camici PG, Baker CS, CW MI (2006). Occurrence of regional left ventricular dysfunction in patients undergoing standard and biofeedback dialysis. Am J Kidney Dis.

[21] McIntyre CW, John SG, Jefferies H (2008). Advances in the cardiovascular assessment of patients with chronic kidney disease. Clin Kidney J.

