# Supplementary material for: Hemodynamic response to non-pneumatic anti-shock compression garments in patients with renal dysfunction
Source: BMC Nephrol. 2020 Jan 14;21:15. doi: 10.1186/s12882-019-1680-8 (PMC6958707; doi:10.1186/s12882-019-1680-8)
Supplement: Supplementary file 1 — Additional file 1: Table S1. Study participants fluid balance as estimated by change from ideal weight. CKD participants (C) and dialysis participants (D) were weighted on the day of study. This weight was compared to their ideal weight, obtained from their recent nephrology clinic notes. The difference between study weight and ideal weight is intended to provide an estimate to the fluid balance of the study participant on the day of study. [file 12882_2019_1680_MOESM1_ESM.docx]

**Table S1.** Study participants fluid balance as estimated by change from ideal weight. CKD participants (C) and dialysis participants (D) were weighted on the day of study. This weight was compared to their ideal weight, obtained from their recent nephrology clinic notes. The difference between study weight and ideal weight is intended to provide an estimate to the fluid balance of the study participant on the day of study.

| Participant | Study Weight | Ideal Weight | ∆ |
| --- | --- | --- | --- |
| C1 | 105.9 | 106 | -0.1 |
| C2 | 82 | 82 | 0 |
| C3 | 116.3 | 116 | 0.3 |
| C4 | 65.9 | 67.5 | -1.6 |
| C5 | 73.6 | 74 | -0.4 |
| D1 | 87.4 | 79 | 8.4 |
| D2 | 67 | 67 | 0 |
| D3 | 71 | 68.5 | 2.5 |
| D4 | Not recorded | 63.5 | N/A |
| D5 | 83 | 82.5 | 0.5 |
